# A Novel Multimodal Radiomics Model for Predicting Prognosis of Resected Hepatocellular Carcinoma

**DOI:** 10.3389/fonc.2022.745258

**Published:** 2022-03-07

**Authors:** Ying He, Bin Hu, Chengzhan Zhu, Wenjian Xu, Yaqiong Ge, Xiwei Hao, Bingzi Dong, Xin Chen, Qian Dong, Xianjun Zhou

**Affiliations:** ^1^ Department of Pediatric Surgery, The Affiliated Hospital of Qingdao University, Qingdao, China; ^2^ Department of Radiology, The Affiliated Hospital of Qingdao University, Qingdao, China; ^3^ Department of Hepatobiliary and Pancreatic Surgery, The Affiliated Hospital of Qingdao University, Qingdao, China; ^4^ GE Healthcare, Shanghai, China; ^5^ Shandong Key Laboratory of Digital Medicine and Computer Assisted Surgery, The Affiliated Hospital of Qingdao University, Qingdao, China; ^6^ Shandong College Collaborative Innovation Center of Digital Medicine Clinical Treatment and Nutrition Health, Qingdao University, Qingdao, China

**Keywords:** liver cancer, multimodal imaging, computed tomography, MRI, radiomics, nomogram

## Abstract

**Objective:**

To explore a new model to predict the prognosis of liver cancer based on MRI and CT imaging data.

**Methods:**

A retrospective study of 103 patients with histologically proven hepatocellular carcinoma (HCC) was conducted. Patients were randomly divided into training (n = 73) and validation (n = 30) groups. A total of 1,217 radiomics features were extracted from regions of interest on CT and MR images of each patient. Univariate Cox regression, Spearman’s correlation analysis, Pearson’s correlation analysis, and least absolute shrinkage and selection operator Cox analysis were used for feature selection in the training set, multivariate Cox proportional risk models were established to predict disease-free survival (DFS) and overall survival (OS), and the models were validated using validation cohort data. Multimodal radiomics scores, integrating CT and MRI data, were applied, together with clinical risk factors, to construct nomograms for individualized survival assessment, and calibration curves were used to evaluate model consistency. Harrell’s concordance index (C-index) values were calculated to evaluate the prediction performance of the models.

**Results:**

The radiomics score established using CT and MR data was an independent predictor of prognosis (DFS and OS) in patients with HCC (*p* < 0.05). Prediction models illustrated by nomograms for predicting prognosis in liver cancer were established. Integrated CT and MRI and clinical multimodal data had the best predictive performance in the training and validation cohorts for both DFS [(C-index (95% CI): 0.858 (0.811–0.905) and 0.704 (0.563–0.845), respectively)] and OS [C-index (95% CI): 0.893 (0.846–0.940) and 0.738 (0.575–0.901), respectively]. The calibration curve showed that the multimodal radiomics model provides greater clinical benefits.

**Conclusion:**

Multimodal (MRI/CT) radiomics models can serve as effective visual tools for predicting prognosis in patients with liver cancer. This approach has great potential to improve treatment decisions when applied for preoperative prediction in patients with HCC.

## Introduction

Hepatocellular carcinoma (HCC) is the most common primary liver tumor, accounting for 75%–85% of liver cancers (1). HCC is the second most common cause of cancer death worldwide and has high morbidity and mortality rates (2). Surgical resection and local ablation remain the most commonly used radical treatment methods for HCC; however, tumors recur in 70% of cases after hepatectomy and 25% of cases after liver transplantation, and the 5-year overall survival (OS) rate is only approximately 25%–55% (3–5). Hence, patients with HCC have a poor prognosis after surgery, and the high disease recurrence rate represents a great challenge to successful treatment (3, 6). Therefore, the identification of reliable predictors of early recurrence is critical for patient risk stratification, support for treatment decisions, and improvement of long-term survival.

At present, relevant tumor factors, such as lesion diameter, cirrhosis, multifocality, poorly differentiated tumor, and microvascular invasion (MVI), are recognized as risk factors for early disease recurrence (7–10); however, most of these features can only be evaluated by postoperative histopathological examination, which is invasive, and the results are prone to a missed diagnosis. In oncology, the application of radiomics, which involves the transformation of traditional medical images into high-dimensional, quantitative, and exploitable imaging data, enables in-depth characterization of tumor phenotypes and has the potential to provide information on intra-tumor heterogeneity and predict posttreatment survival (11, 12). Multimodal machine learning is a method to process and interpret multimodal information through machine learning. Multimodal fusion is used to fuse multimodal information and perform targeted prediction classification or regression problems (13–15). Medical imaging can include data in different forms, such as CT, MRI, PET, ultrasound, and X-rays. In different guidelines, either CT or MRI is proposed as the best imaging modality for the diagnosis of HCC (16–18). Recent HCC management guidelines recognize an increasing role for gadoxetic acid-enhanced MRI in early diagnosis and monitoring post-resection (19). CT or MRI can all confirm the diagnosis if a nodule larger than 1-cm diameter is found with typical vascular features of HCC (hypervascularity in the arterial phase with washout in the portal venous or delayed phase) (20). Further, both CT and MR functional scans can be useful as supplements to conventional plain scan and dynamic enhancement to improve the accuracy of follow-up evaluation of liver cancer (21). In recent years, several qualitative MRI and CT imaging features have been reported. Preliminary evidence suggests that radiomics features have the potential to predict OS and tumor recurrence in patients with HCC, for example, by assessing peritumor parenchymal enhancement, satellite nodules, and non-smooth tumor margins, which are non-invasive predictors of early HCC recurrence (22–24).

Multimodal fusion technology can be divided into pixel level, feature level, and decision level, which are used to fuse abstract features and decision results in original data (13–15). To date, radiomics has been successfully applied in the study of nasopharyngeal carcinoma, non-small cell lung cancer, and rectal cancer (25–27), demonstrating the great potential for the development of this approach; however, to our knowledge, the use of contrast analysis of CT-enhanced sequence and MR-enhanced sequence data to assess patient prognosis remains rare. In this study, we combined these two novel imaging techniques and explored the performance of multimodal radiomics models derived from MR and CT image data for prognostic evaluation following HCC resection.

## Materials and Methods

### Patients

This study was approved by the Ethics Committee of the Affiliated Hospital of Qingdao University. Due to its retrospective nature, the need for patient written informed consent was waived. From February 2014 to December 2020, we collected information from 306 patients with liver cancer, and 135 patients with primary HCC were recruited, based on the following inclusion criteria: 1) pathologically confirmed liver cancer recorded in the medical records at our hospital and 2) CT and MRI examinations performed within the previous 2 weeks before hepatectomy. The exclusion criteria were as follows: 1) other preoperative treatments [transarterial chemoembolization (TACE)], targeted drugs, and radiofrequency ablation), except hepatectomy (n = 11); 2) incomplete clinicopathological report (n = 10); 3) CT image and MR image quality was poor, and the lesion could not be recognized or the lesion image was less than three layers (n = 3); 4) lost to follow-up (n = 4); and 5) error occurred in the feature extraction process (n = 4). The final study population included 103 patients. The entire cohort was randomly divided into a training cohort (n = 73) and a validation cohort (n = 30) (ratio, 7:3). Training queues were used to build single-modal and multimodal radiomics models, which were evaluated using validation queues.

### Clinical Endpoints and Follow-Up

The endpoints of this study were disease-free survival (DFS) and OS. DFS was measured from the date of surgery until disease progression, death from any cause, or the last visit in follow-up (censored), and nomograms were also built based on the DFS. Disease progression, including local recurrence distant metastasis, was confirmed by clinical examination and imaging methods such as abdominopelvic CT or MRI or was biopsy-proven. OS was defined as the time to death from any cause. All patients were followed up after surgery. Serum alanine transaminase (ALT), aspartate transaminase (AST), total bilirubin (TBIL), albumin (ALB), and alpha-fetoprotein (AFP) levels were obtained. Liver ultrasound examination was performed monthly within the 3 months after surgery and once every 3 months thereafter. CT examination of the lungs and enhanced CT or MRI of the liver were performed every 3 months during the first 2 years and once every 6 months thereafter. The minimum follow-up period was 3 days after surgery, while the maximum follow-up time was 92.8 months.

### Image Acquisition

#### CT Scanning Methods and Parameters

Three-stage enhanced scans of the upper abdomen were obtained using a German CT (SOMATOM Definition Flash, Siemens, Munich, Germany) and an American Discovery CT (GE Healthcare, Chicago, IL, USA). Scans ranged from the top of the liver to the lower edges of both kidneys. Scanning parameters were as follows: voltage, 120 kV; current, 200–350 mA; scanning layer thickness, 5 mm; layer spacing, 5 mm; and matrix, 512 × 512. For contrast-enhanced scanning, a double-barreled high-pressure syringe was used to inject iohexol, containing 350 mg/ml of iodine, *via* the peripheral vein (flow rate, 3.0 ml/s; dose, 1.5 ml/kg). The delay times for the arterial, venous, and equilibrium phases were 30, 60, and 120 s, respectively.

#### MRI Scanning Methods and Parameters

MRI scanning was conducted using a 3.0 T Signa HDXT MR superconducting apparatus and an 8-channel body-phase front coil. Rapid volume acquisition Liver Acquisition with Volume Acceleration (LAVA) imaging of the liver was conducted using the following parameters: repetition time (TR), 4.2 ms; echo time (TE), 2.0 ms; layer thickness, 4.8–5.4 mm; layer spacing, 1.4–2.7 mm; field, 42.0 × 33.6 cm; and matrix, 320 × 192. The contrast agent, gadolinium diethylenetriamine penta-acetic acid, was used for enhanced scanning (dose, 0.2 mmol/kg; injection flow rate, 2.0 ml/s). The delay times of the arterial, portal, and equilibrium phases were 20–23, 60, and 180 s, respectively.

### Tumor Segmentation

The tumor region of interest (ROI) was manually delineated on multi-phase CT and MR images by a radiologist with more than 10 years of experience (Reader 1) using ITK-SNAP (version 3.6.0; http://www.itksnap.org) to segment each tumor CT stage and MR stage. A two-dimensional ROI of the largest section of the tumor was selected, outlined, and saved as an NII file. Two weeks later, Reader 1 randomly selected 50 HCC patients and delineated the ROI again to evaluate the intra-class correlation coefficient of ROI. Additionally, another radiologist (Reader 2) independently performed ROI mapping for the randomly selected 50 HCC patients to evaluate the inter-class correlation coefficient.

### Image Preprocessing and Feature Extraction

At the beginning of extraction, pre-processing was necessary to improve discrimination between texture features. To eliminate the batch effect of different equipment, all the data were normalized through z-score standardization to a standard intensity range with a mean value of 0 and SD of 1, and the image slices were resampled to voxel size = 1 * 1 * 1 cm^3^. With the use of IBSI compliant AK software (Analysis Kit Software, version 3.3.0, GE Healthcare), 1,217 radiomics features were extracted from CT and MR images, including first-order statistical features, morphological features, gray-level co-occurrence features, matrix-based features (GLCM), gray-level run-length matrix-based features (GLRLM), gray-level size zone matrix-based features (GLSZM), gray-level dependence matrix-based features (GLDM), and (Log) Laplace wavelet changes. Furthermore, intra-class and inter-class correlation coefficients (ICCs) were used to evaluate the intra-observer and inter-observer reproducibility of feature extraction. The intra-class correlation coefficient was calculated by comparing the ROI of Reader 1 twice. The inter-class correlation coefficient between the groups was evaluated by comparing the ROI of Reader 1 with that of Reader 2. When ICCs exceeded 0.75 both within and between observers, this feature was considered to have a good consistency. Finally, the ICC range for CT (Balance, Venous, and Artery) was 0.175–1, and 917 features with ICC > 0.75 were retained for each phase. The ICC range for MR (Balance, Venous, and Artery) was 0.256–1, and 946 features with ICC > 0.75 were retained.

### Feature Selection and Model Construction

Features with ICC values > 0.75 both within and between groups were retained for further analysis. In the training set, features with *p* < 0.05 in univariate Cox regression analysis were retained, and Spearman’s correlation analysis and Pearson’s correlation analysis were applied to eliminate characteristics that were highly correlated (selected coefficient threshold |r| = 0.8). The least absolute shrinkage and selection operator (LASSO) Cox regression with 10-fold cross-validation was used for further feature screening. Then, features with non-zero coefficients selected by LASSO analysis were linearly weighted. Next, radiomics scores (Radscores) were calculated for each patient. The Radscore was the result of the Cox regression radiomics model. It was the linear combination weighted by the corresponding LASSO coefficients of each feature selected of each patient, and patients were then divided into high-risk and low-risk groups, according to their best truncation value in each model and the labeled high-risk group (riskscore = 1) and the low-risk group (riskscore = 0). Kaplan–Meier (KM) analysis was used to plot DFS and OS curves, and the log-rank test was used to evaluate the differences between high-risk and low-risk groups. The same threshold was then applied to the validation queue. C-index values were used to evaluate the performance of the model.

### Nomogram Construction

First, univariate Cox analysis was used to analyze risk factors and screen for features with *p* < 0.05. Clinical factors with *p* < 0.05 and Radscore for CT and MRI data combined (Combined_radscore) were included in the multivariate Cox stepwise regression model, to investigate independent predictors of survival in HCC patients. Clinical factors and Combined_radscore (with *p* < 0.05) in the univariate Cox analysis were enrolled to establish a nomogram to predict patients’ 2-year, 4-year, and 5-year survival rates. C-index values were used to evaluate the performance of the model, and calibration curves were generated and discrimination ability was quantified to compare predicted and actual survival rates.

### Statistical Analysis

All statistical analyses were performed using R3.5.1 (https://www.r-project.org/). A t-test or Mann–Whitney U test was used to evaluate differences in continuous variables, and the chi-square or Fisher’s exact test to assess differences in categorical variables. Continuous numerical variables are represented by the median (25th percentile, 75th percentile), and categorical variables are represented by percentages. Shapiro’s test function in the R package was used to test for normality. Spearman’s correlation analysis and Pearson’s correlation analysis were used to eliminate redundant features. Pearson’s correlation analysis was used for the features that conform to the normal distribution, and Spearman’s correlation analysis was used for the features that do not conform to normal distribution. The surv_cutpoint function in the R package was used to calculate optimal truncation values. The KM method and log-rank test were used to estimate DFS and OS. Calibration curves were used to evaluate the degree of alignment of nomograms. Two-sided *p*-values <0.05 were considered significant.

## Results

### Patient Characteristics

Patient demographics and clinicopathological features are presented in **Table 1**. Of the 103 patients included in the study, 83 (80.6%) were male, and the median age of all patients was 57.0 (32.0–73.0) years. There were no statistically significant differences in clinicopathological factors between patients in the training (n = 73, 70%) and validation (n = 30, 30%) cohorts (*p* = 0.558–0.997). A total of 44 patients had death endpoints. The median values for DFS and OS of the total patient group (n = 103) were 25.9 (0.1–88.1) months and 43.7 (0.1–92.8) months, respectively.

**Table 1:** Demographic and clinicopathological characteristics of patients with liver cancer.

Variable		Training cohort (N = 73)	Validation cohort (N = 30)	*p*
Age (years),	>60	32 (0.44)	12 (0.40)	0.721
	≤60	41 (0.56)	18 (0.60)	
Gender	Male	60 (0.82)	7 (0.23)	0.520
	Female	13 (0.18)	23 (0.77)	
Alcohol abuse (%)	Present	13 (0.18)	6 (0.20)	0.794
	Absent	60 (0.82)	24 (0.80)	
AFP (ng/ml, %)	≤20	32 (0.44)	11 (0.37)	0.503
	>20	41 (0.56)	19 (0.63)	
HBV (%)	Present	63 (0.86)	23 (0.77)	0.231
	Absent	10 (0.14)	7 (0.23)	
HBsAg (%)	Positive	62 (0.85)	23 (0.77)	0.316
	Negative	11 (0.15)	7 (0.23)	
Pos_operation_TACE (%)	Present	29 (0.40)	10 (0.33)	0.543
	Absent	44 (0.60)	20 (0.67)	
Tumor diameter (cm, %)	≤5 cm	52 (0.71)	17 (0.57)	0.153
	>5 cm	21 (0.29)	13 (0.43)	
Tumor number (%)	≥2	8 (0.11)	3 (0.10)	0.835
	<2	65 (0.89)	27 (0.9)	
MVI (%)	Present	35 (0.48)	17 (0.57)	0.421
	Absent	38 (0.52)	13 (0.43)	
PV-TT (%)	Present	3 (0.04)	2 (0.07)	0.627
	Absent	70 (0.96)	28 (0.93)	
Satellite lesions (%)	Present	9 (0.12)	3 (0.10)	0.997
	Absent	64 (0.88)	27 (0.90)	
Liver cirrhosis (%)	Present	61 (0.84)	26 (0.87)	0.924
	Absent	12 (0.16)	4 (0.13)	
Surgical margin (%)	<1 cm	26 (0.36)	16 (0.53)	0.094
	≥1 cm	47 (0.64)	14 (0.47)	
Liver capsule invasion (%)	Present	39 (0.53)	13 (0.43)	0.352
	Absent	34 (0.47)	17 (0.57)	
Surgical approach (%)	Laparoscopy	22 (0.30)	10 (0.33)	0.750
	Non-laparoscopy	51 (0.70)	20 (0.67)	
Histopathological grading	I, II	41 (0.56)	16 (0.53)	0.793
	III, IV	32 (0.44)	14 (0.47)	
Child–Pugh score (%)	A	71 (0.97)	26 (0.87)	0.058
	B	2 (0.03)	4 (0.13)	
CNLC (%)	I, II	66 (0.90)	25 (0.83)	0.309
	III, IV	7 (0.10)	5 (0.17)	
Bleeding_volume (ml, %)	≤400	64 (0.88)	27 (0.90)	0.997
	>400	9 (0.12)	3 (0.10)	
BMI (kg/m^2^)		25.28 (22.67, 26.57)	23.81 (21.87, 25.69)	0.209
ALT (IU/L)		38 (21, 69)	40.50 (26.50, 97.93)	0.408
AST (IU/L)		29 (21, 57)	31.5 (22.25, 64, 35)	0.452
TBIL (µmol/L)		17.07 (13.56–22.50)	18.31 (13.61, 25.65)	0.338
ALB (g/L)		40.05 (37.29, 43.41)	40.71 (37.25, 43.96)	0.836
PT (s)		10.5 (9.80, 11.10)	10.60 (9.83, 11.17)	0.825
PLT (10^9^/L)		160 (127, 209)	164 (116, 190)	0.554
NEUT (10^9^/L)		2.97 (2.12, 4.74)	3.51 (2.88, 4.52)	0.200
Lymphocyte (10^9^/L)		1.9 (1.36, 3.77)	1.71 (1.43, 2.57)	0.862

BMI, body mass index; AFP, alpha-fetoprotein; HBsAg, hepatitis B surface antigen status; MVI, microvascular invasion; PV-TT, portal vein tumor thrombosis; CNLC, China Liver Cancer Staging; ALT, alanine aminotransferase; AST, aspartate aminotransferase; TBIL, total bilirubin; ALB, albumin; PT, prothrombin time; PLT, platelet count; NEUT, neutrophil count.

### Radiomics Signature Construction

Features retained after each feature dimension reduction are listed in **Supplementary Table S1**. Finally, for prediction of DFS, 7, 12, and 17 features were selected from CT, MRI, and their combined features, respectively, and used to build models. For prediction of OS, 8, 16, and 17 features were selected to establish the model from CT, MRI, and their combined features, respectively. The details of selected features of DFS and OS are included in **Supplementary Figure S1** and **Table S2**. The calculated CT_radscore, MRI_radscore, and Combined_radscore were based on selected features.

We performed the univariate Cox analysis to determine the role of clinical features of patients on DFS in HCC (**Table 2**). Three clinical characteristics, namely, tumor diameter, liver capsule invasion, and MVI were identified by univariate analysis (*p* < 0.05). Clinical features with *p* < 0.05 were included in backward stepwise multivariate regression analysis. The results show that MVI was an independent predictor of HCC in the multivariable analysis (*p* < 0.05). We performed the univariate Cox analysis to determine the role of clinical characteristics on the OS of patients in HCC (**Table 3**). Six clinical characteristics, namely, body mass index (BMI), tumor diameter, MVI, portal vein tumor thrombosis (PV_TT), platelet count (PLT), and Bleeding_volume were identified by univariate analysis (*p* < 0.05). Clinical characteristics with *p* < 0.05 were included in backward stepwise multivariate regression analysis. The results show that BMI, MVI, and Bleeding_volume were independent predictors of HCC in the multivariable analysis (*p* < 0.05). The clinical models were built based on clinical risk features, and the Clinical_score of each model was calculated.

**Table 2:** Univariate and multivariate analyses of training cohort to identify patient clinical features with prognostic value for DFS.

Variable	Univariate analysis		Multivariate analysis	
	HR (95% CI)	*p*-Value	HR (95% CI)	*p*-Value
Age	0.994 (0.961–1.028)	0.708		
Gender	1.712 (0.723–4.055)	0.222		
BMI	1.014 (0.992–1.036)	0.229		
Alcohol	1.088 (0.506–2.342)	0.829		
Liver cirrhosis	1.436 (0.607–3.399)	0.410		
Histopathological grade	1.361 (0.842–2.199)	0.209		
Tumor diameter	1.128 (1.02–1.247)	<0.05	1.07 (0.96–1.19)	0.244
Liver capsule invasion	1.907 (1.036–3.509)	<0.05	1.41 (0.74–2.72)	0.299
Surgical margin	1.025 (0.963–1.091)	0.445		
Tumor number	1.329 (0.583–3.027)	0.499		
Satellite lesions	1.43 (0.602–3.393)	0.418		
MVI	4.338 (2.31–8.147)	<0.05	3.95 (2.07–7.54)	<0.05
PV_TT	1.412 (0.34–5.867)	0.635		
HBV	0.833 (0.352–1.971)	0.677		
HBsAg	0.999 (0.997–1.003)	0.953		
Surgical approach	1.198 (0.626–2.291)	0.585		
Pos_operation_TACE	1.652 (0.911–2.996)	0.099		
AFP	1.000 (0.999–1.000)	0.547		
PLT	0.999 (0.995–1.004)	0.806		
PT	1.002 (0.989–1.015)	0.749		
Alb	1.014 (0.949–1.084)	0.675		
TBIL	0.954 (0.907–1.003)	0.067		
ALT	1.001 (0.998–1.003)	0.594		
AST	1.001 (0.999–1.003)	0.307		
NEUT	1.088 (0.978–1.209)	0.120		
Lymphocyte	0.987 (0.96–1.016)	0.379		
Bleeding_volume	1.000 (0.999–1.000)	0.201		
Child–Pugh score	0.746 (0.103–5.422)	0.772		
CNLC	0.77 (0.464–1.278)	0.312		

BMI, body mass index; MVI, microvascular invasion; PV-TT, portal vein tumor thrombosis; HBsAg, hepatitis B surface antigen status; TACE, transarterial chemoembolization; AFP, alpha-fetoprotein; PLT, platelet count; PT, prothrombin time; ALB, albumin; TBIL, total bilirubin; ALT, alanine aminotransferase; AST, aspartate aminotransferase; NEUT, neutrophil count; CNLC, China Liver Cancer Staging; HR, hazard ratio.

**Table 3:** Univariate and multivariate analyses of training cohort to identify patient clinical features with prognostic value for OS.

Variable	Univariate analysis		Multivariate analysis	
	HR (95% CI)	*p*-Value	HR (95% CI)	*p*-Value
Age	1.018 (0.98–1.057)	0.351		
Gender	0.484 (0.215–1.092)	0.081		
BMI	0.881 (0.798–0.972)	<0.05	0.850 (0.740–0.970)	<0.05
Alcohol	0.92 (0.378–2.240)	0.853		
Liver cirrhosis	0.952 (0.383–2.367)	0.916		
Histopathological grade	1.695 (0.965–2.977)	0.066		
Tumor diameter	1.188 (1.063–1.327)	<0.05	1.100 (0.910–1.320)	0.329
Liver capsule invasion	1.853 (0.888–3.867)	0.100		
Surgical margin	1.053 (0.991–1.120)	0.096		
Tumor number	0.947 (0.419–2.139)	0.895		
Satellite lesions	1.136 (0.339–3.805)	0.836		
MVI	6.935 (2.962–16.239)	<0.05	5.060 (2.080–12.310)	<0.05
PV_TT	3.87 (1.142–13.114)	<0.05	3.190 (0.870–11.650)	0.079
HBV	0.555 (0.212–1.454)	0.231		
HBsAg	0.998 (0.994–1.001)	0.155		
Surgical approach	1.267 (0.599–2.680)	0.535		
Pos_operation_TACE	1.305 (0.641–2.658)	0.463		
AFP	1.000 (0.999–1.000)	0.136		
PLT	0.993 (0.986–1.000)	<0.05	0.990 (0.990–1.000)	0.174
PT	1.003 (0.982–1.024)	0.812		
Alb	1.003 (0.932–1.080)	0.937		
TBIL	0.989 (0.952–1.028)	0.579		
ALT	0.999 (0.995–1.002)	0.500		
AST	0.998 (0.994–1.003)	0.478		
NEUT	1.07 (0.900–1.273)	0.442		
Lymphocyte	0.975 (0.936–1.015)	0.219		
Bleeding_volume	1.001 (1.001–1.002)	<0.05	1.000 (1.000–1.010)	<0.05
Child–Pugh score	1.784 (0.237–13.428)	0.574		
CNLC	1.313 (0.787–2.190)	0.298		

BMI, body mass index; MVI, microvascular invasion; PV-TT, portal vein tumor thrombosis; HBsAg, hepatitis B surface antigen status; TACE, transarterial chemoembolization; AFP, alpha-fetoprotein; PLT, platelet count; PT, prothrombin time; ALB, albumin; TBIL, total bilirubin; ALT, alanine aminotransferase; AST, aspartate aminotransferase; NEUT, neutrophil count; CNLC, China Liver Cancer Staging; HR, hazard ratio.

Combined_radscore and clinical factors were included in univariate Cox regression for analyzing DFS, and factors with *p* < 0.05 were included in backward stepwise multivariate Cox regression analysis (**Table 4**). The results show that Radscore and MVI were independent predictors of HCC in the multivariable analysis (*p* < 0.05). Combined_radscore and clinical factors were included in univariate Cox regression for analyzing OS, and factors with *p* < 0.05 were included in backward stepwise multivariate Cox regression analysis (**Table 5**). The results show that Radscore, MVI, PLT, and Bleeding_volume were independent predictors of HCC in the multivariable analysis (*p* < 0.05). CT+MRI_Clinical Model was established based on significant clinical risk features and Radscore. CT+MRI+Clinical_score of the models were calculated.

**Table 4:** Univariate and multivariate analyses of training cohort to identify patient clinical features and Combined_radscore with prognostic value for DFS.

Variable	Univariate analysis		Multivariate analysis	
	HR (95% CI)	*p*-Value	HR (95% CI)	*p*-Value
Tumor diameter	1.128 (1.020–1.247)	<0.05	1.290 (0.660–2.520)	0.456
Liver capsule invasion	1.907 (1.036–3.509)	<0.05	0.970 (0.870–1.080)	0.593
MVI	4.338 (2.310–8.147)	<0.05	3.090 (1.520–6.310)	<0.05
Radscore	6.553 (3.975–10.803)	<0.05	5.600 (3.340–9.370)	<0.05

DFS, disease-free survival; MVI, microvascular invasion; Radscore, radiomics score; HR, hazard ratio.

**Table 5:** Univariate and multivariate analyses of training cohort to identify patient clinical features and Combined_radscore with prognostic value for OS.

Variable	Univariate analysis		Multivariate analysis	
	HR (95% CI)	*p-*Value	HR (95% CI)	*p*-Value
BMI	0.881 (0.798–0.972)	<0.05	0.970 (0.880–1.060)	0.480
Tumor diameter	1.188 (1.063–1.327)	<0.05	0.840 (0.660–1.080)	0.174
MVI	6.935 (2.962–16.239)	<0.05	4.110 (1.550–10.87)	<0.05
PV_TT	3.870 (1.142–13.114)	<0.05	2.030 (0.510–8.160)	0.318
PLT	0.993 (0.986–1.000)	<0.05	0.990 (0.980–1.000)	<0.05
Bleeding_volume	1.001 (1.001–1.002)	<0.05	1.000 (1.000–1.010)	<0.05
Radscore	6.959 (3.922–12.349)	<0.05	7.740 (3.560–16.800)	<0.05

OS, overall survival; BMI, body mass index; MVI, microvascular invasion; PV-TT, portal vein tumor thrombosis; platelet count; Radscore, radiomics score; HR, hazard ratio.

**Figure 1:**
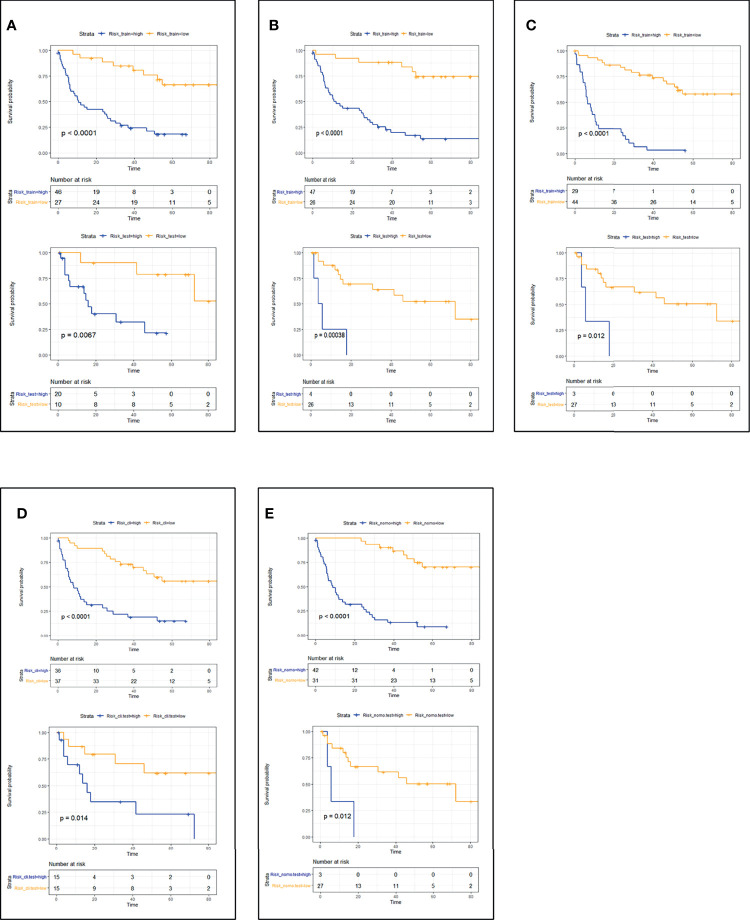
Patient DFS KM curves for each model. **(A)** CT_DFS; **(B)** MRI_DFS; **(C)** CT+MRI_DFS; **(D)** Clinical_DFS; **(E)** CT+MRI+Clinical_DFS. *p*-Values were calculated using the log-rank test. Training cohort curves are shown on the top and validation cohorts on the bottom in each panel. DFS, disease-free survival; KM, Kaplan–Meier.

**Figure 2:**
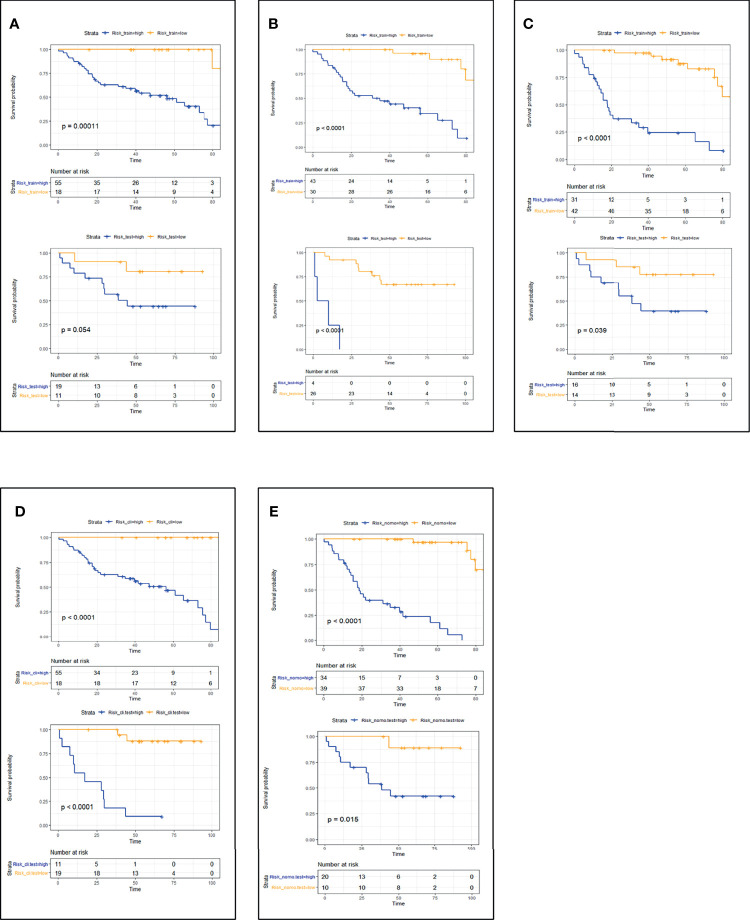
Patient OS KM curves for each model: **(A)** CT_OS; **(B)** MRI_OS; **(C)** CT+MRI_OS; **(D)** Clinical_OS; **(E)** CT+MRI+Clinical_OS. *p*-Values were calculated using the log-rank test. Training cohort curves are shown on the top and validation cohorts on the bottom in each panel. OS, overall survival; KM, Kaplan–Meier.

CT_radscore, MRI_radscore, Combined_radscore, Clinical_score, and CT+MRI+Clinical_score were divided into a high-risk group and a low-risk group according to the optimal cutoff value of each group, and then DFS and OS KM curves were plotted. KM curves methods and log-rank test estimating DFS (**Figure 1**) in the training cohort showed that patients in the low-risk group had significantly better outcomes than those in the high-risk group (all log-rank *p* < 0.05) using the model. We then performed the same analyses in the validation cohort. Each model had similar results in the validation cohort (*p* < 0.05). KM curves methods and log-rank test estimating OS (**Figure 2**) in the training cohort showed that patients in the low-risk group had significantly better outcomes than those in the high-risk group (*p* < 0.05). We then performed the same analyses in the validation cohort, and similar results were observed.

### Development and Assessment of a Radiomics Nomogram

**Figure 3:**
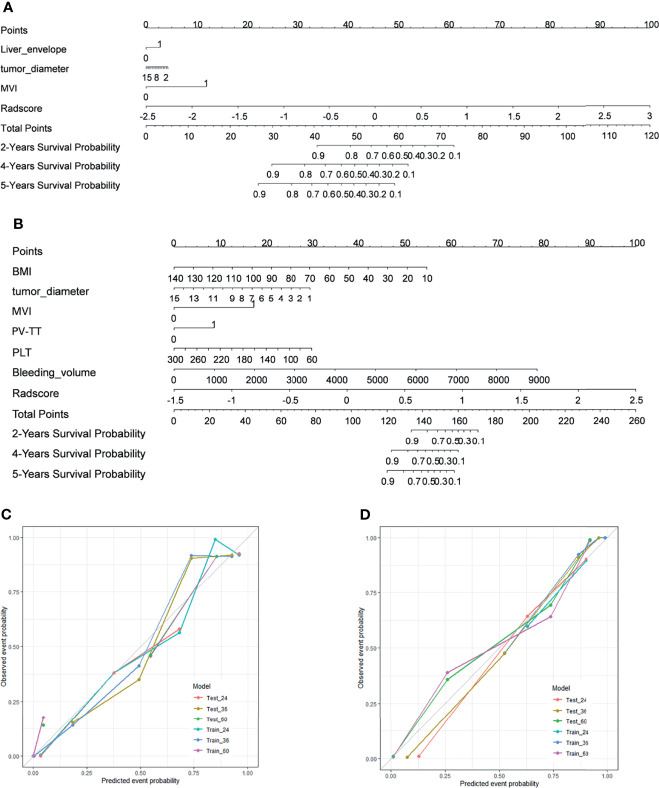
Development of nomograms and calibration curves for DFS and OS in training cohorts. **(A)** Prognostic nomogram for DFS. **(B)** The prognostic nomogram for OS. **(C)** Calibration curves for DFS in the training cohort. **(D)** Calibration curves for OS in the training cohort. To determine the number of factors associated with the probability of survival, a straight line was drawn to the relevant point on the axis for each patient, and the process was repeated for each variable. Scores for each risk factor were then summarized, with the final sum marked on the overall point axis. DFS and OS estimated using the nomogram are plotted on the x-axis. Observed DFS or OS are plotted on the y-axis, and the estimated results are compared with the actual results. The consistency of estimated and observed calibrations for 2-year, 4-year, and 5-year survival results is shown for each model. DFS, disease-free survival; OS, overall survival.

To provide the clinician with a quantitative method to predict patients’ probability of 2-year, 4-year, and 5-year DFS and OS and to demonstrate the incremental value of the radiomics signature for individualized assessment of DFS and OS, both radiomics nomograms were built in the training cohort (**Figures 3A, B**).

For prediction of DFS, Radscore, tumor diameter, liver capsule invasion, and MVI were finally retained to establish a nomogram for DFS prediction (**Figure 3A**), and BMI, tumor diameter, PV_TT, PLT, Bleeding_volume, and Radscore were retained for use in establishing the prognostic prediction nomogram for OS (**Figure 3B**). The performance of each modal for predicting DFS and OS was evaluated by calculating C-index values (**Table 6**). In DFS analysis, the CT+MRI+Clinical model showed the best performance in the training cohort (C-index = 0.858; 95% CI, 0.811–0.905), followed by the CT+MRI model (C-index = 0.826; 95% CI, 0.767–0.885). The clinical model had the lowest predictive performance of C-index = 0.717 (95% CI, 0.648–0.786). In the validation cohort, the CT+MRI+Clinical model showed the best performance (C-index = 0.704; 95% CI, 0.563–0.845), followed by the clinical model (C-index = 0.657; 95% CI, 0.504–0.809). The MRI model had the lowest predictive performance of C-index = 0.587 (95% CI, 0.412–0.763).

**Table 6:** The performance of each model in the training and validation cohorts.

	Model	Training cohort		Validation cohort	
Disease-free survival		C-index	95% CI	C-index	95% CI
	CT	0.742	0.668–0.816	0.614	0.442–0.786
	MRI	0.772	0.705–0.839	0.587	0.412–0.763
	CT+MRI	0.826	0.767–0.885	0.653	0.490–0.816
	Clinical	0.717	0.648–0.786	0.657	0.504–0.809
	CT+MRI+Clinical	0.858	0.811–0.905	0.704	0.563–0.845
Overall survival	CT	0.740	0.650–0.830	0.624	0.450–0.789
	MRI	0.833	0.768–0.898	0.601	0.401–0.801
	CT+MRI	0.865	0.810–0.920	0.653	0.471–0.835
	Clinical	0.802	0.714–0.890	0.705	0.597–0.803
	CT+MRI+Clinical	0.893	0.846–0.940	0.738	0.575–0.901

For analysis of OS, CT+MRI+Clinical had the best predictive performance (C-index = 0.893; 95% CI, 0.846–0.940) in the training cohort, followed by the CT+MRI model (C-index = 0.865; 95% CI, 0.810–0.920); the CT model had the lowest predictive performance (C-index = 0.740; 95% CI, 0.650–0.830).

In the validation cohort, CT+MRI+Clinical had the best predictive performance (C-index = 0.738; 95% CI, 0.575–0.901), followed by the clinical model (C-index = 0.705; 95% CI, 0.597–0.803). The MRI model had the lowest predictive performance of C-index = 0.601 (95% CI, 0.401–801). The calibration curve showed the high accuracy of the nomograms for predicting DFS and OS both in the training dataset (**Figures 3C** **, D**).

## Discussion

Previous studies have developed multimodal imaging models, using radiomics features determined by MR and CT to predict tumor prognosis (28). To our knowledge, the present study is the first to evaluate DFS and OS in patients with HCC using a contrastive learning analysis of enhanced CT and MRI sequence data. The main challenges faced by multi-pattern methods are how to judge the confidence of each mode and the correlation between modes, how to reduce the dimension of multi-pattern characteristic information, and how to register multi-pattern data collected asynchronously (13–15). We compared the advantages of multimodal radiomics models for CT and MRI integration.

Radiomics has recently received attention in the field of cancer research because it is a high-throughput method used to extract large numbers of radiomics features from standard medical imaging and can improve medical decisions (29). Radiomics is used to extract quantitative feature data that reflect information related to tumor heterogeneity, which are not visible to the human eye. Hence, radiomics can provide a non-invasive, low-cost, and reproducible means to capture tumor phenotypes that may be associated with intra-tumor heterogeneity (30). To date, radiomics has been used in research to explore liver tumors, including numerous studies applied to the diagnosis, prognosis, pathological grading, and MVI of liver cancer (31–34). Many previous studies have demonstrated the role of radiomics in survival assessment for patients with different types of cancer, including non-small cell lung, breast, and thyroid cancers (35–37).

We developed a new multimodal radiomics model to compare the value of enhanced CT and MRI sequence data for prognosis prediction in patients with HCC and to compare this with the predictive performance of clinicopathological factors. In this study, we extracted 1,217 features from CT and MR images and finally identified non-zero coefficient features associated with DFS and prognostic features associated with OS by LASSO regression analysis. Specific feature dimension reduction and features screening processes are also shown in the Supplementary Materials. Radscore values were calculated using these features. KM survival analysis methods and log-rank tests were used to evaluate their prognostic value.

In our study, the results of multivariate analyses showed that MVI, Bleeding_volume, and PLT were independent predictors of the prognosis of HCC patients, which was consistent with the results of previous studies (7–10). The CT+MRI+Clinical model was superior to that of a model comprising clinical features alone, CT alone, MRI alone, or CT+MRI combined model, indicating that the multimodal radiomics model approach may have a greater value in predicting DFS and OS of resected HCC. The multimodal model can provide more abundant information.

In addition, for all KM curves of predicting DFS and OS, the low-risk group had significantly higher survival times than the high-risk group (*p* < 0.05), indicating that Radscore was an independent predictor of HCC, and this finding was confirmed in the multivariate Cox proportional risk model (*p* < 0.05) in both DFS and OS. Thus, Radscore improves traditional prognostic ability and represents a potentially effective and promising tool for evaluating the prognosis of patients with HCC. This is consistent with the study by Zhao et al. (38). In a prior study, Zhang et al. (28) established single and multimodal logic models for predicting LVI, with excellent predictive power in training (area under the curve (AUC), 0.884; 95% CI, 0.803–0.964) and validation (AUC, 0.876; 95% CI, 0.721–1.000). Their results are similar to our study, but our model also included clinical factors. Univariate and multivariate Cox analyses were used to select clinical factors into the model to analyze the prognosis, which was more convincing and scientific by comparing the prediction performance of various modes, and it was shown in nomograms. Our Radscore-based nomograms yielded a better discriminative ability than these traditional methods for predicting prognosis in HCC patients.

Zhou et al. (24, 38) extracted radiomics features from arterial and portal phase CT images of 215 HCC patients undergoing partial hepatectomy, screened the imaging features through a LASSO logistic regression model, and constructed a Radscore model. The results showed that inclusion of CT-based radiomics features with routine clinical variables significantly predicted early recurrence (≤1 year) postoperatively and that the diagnostic performance of the model combining radiomics and clinical factors was superior to that of the model with clinical features alone for estimating early recurrence. It seems to be obvious that assessing tumorous disease with single modal radiomics information will not be comprehensive. However, the development of methods and strategies for the integration of information of different dimensions is still in its early stages, and combining prediction models, as performed in the current study, might increase their precision and could be extended to other diagnostic indicators. Further research following this scheme is warranted.

This study has several limitations. First, our study was conducted in a single institution. Although all CT and MR images were obtained using a uniform scanner and standardized imaging acquisition sequences, to reduce bias and variance in our results and improve the robustness of the model, further confirmation using patient data from other institutions is needed. Second, the use of manually drawn two-dimensional ROI is time-consuming and inconvenient for clinical application; hence, the feasibility of automatic segmentation or semi-segmentation in radiomics analysis will be the focus of future research. Third, the number of patients in this study is not large because not all HCC patients need to undergo CT and MR in clinical practice. In addition, the cost of conducting CT and MR at the same time is relatively expensive, so there are some obstacles to implementation. Finally, our single-center study primarily included patients who had undergone CT and MR, with a small sample size. We will work with other hospitals to explore the robustness of similar multimodal models in the future.

In conclusion, our results suggest that Radscore is an independent prognostic factor in patients with HCC. Multimodal imaging profiles have great potential to improve individualized assessment of likely prognosis after surgery and may guide the individualized care of patients with HCC.

## Data Availability Statement

The raw data supporting the conclusions of this article will be made available by the authors, without undue reservation.

## Ethics Statement

The studies involving human participants were reviewed and approved by the Affiliated Hospital of Qingdao University. Written informed consent for participation was not required for this study in accordance with the national legislation and the institutional requirements.

## Author Contributions

YH, CZ, QD, BH, and XZ contributed to the conception and design. YH, BH, WX, and XC organized the database. YG, XH, and BD managed the patient and provided technical support. YH wrote the first draft of the manuscript. YH and YG performed the statistical analysis, CZ, QD, and XZ reviewed and revised the manuscript. All authors listed have made a substantial, direct, and intellectual contribution to the work and approved it for publication.

## Funding

This work was supported by Clinical Medicine + X (grant number 3756).

## Conflict of Interest

YG was employed by GE Healthcare.

The remaining authors declare that the research was conducted in the absence of any commercial or financial relationships that could be construed as a potential conflict of interest.

## Publisher’s Note

All claims expressed in this article are solely those of the authors and do not necessarily represent those of their affiliated organizations, or those of the publisher, the editors and the reviewers. Any product that may be evaluated in this article, or claim that may be made by its manufacturer, is not guaranteed or endorsed by the publisher.

## Abbreviations

HCC, hepatocellular carcinoma; DFS, disease-free survival; OS, overall survival; C-index, Harrell’s concordance index; TACE, transarterial chemoembolization; ALT, alanine aminotransferase; AST, aspartate aminotransferase; TBIL, total bilirubin; ALB, albumin; AFP, alpha-fetoprotein; ROI, region of interest; ICCs, the intra-class coefficient and the inter-class correlation coefficient; GLCM, gray-level co-occurrence features matrix-based features; GLRLM, gray-level run-length matrix-based features; GLSZM, gray-level size zone matrix-based features; GLDM, gray-level dependence matrix-based features; Log, Laplace wavelet; LASSO, least absolute shrinkage and selection operator; KM, Kaplan–Meier; Radscore, radiomics score; MVI, microvascular invasion; BMI, body mass index; PV_TT, portal vein tumor thrombosis; PLT, platelet count; HBsAg, hepatitis B surface antigen status; PT, prothrombin time; NEUT, neutrophil count.
