# Supplementary material for: A Novel Multimodal Radiomics Model for Predicting Prognosis of Resected Hepatocellular Carcinoma
Source: Front Oncol. 2022 Mar 7;12:745258. doi: 10.3389/fonc.2022.745258 (PMC8936674; doi:10.3389/fonc.2022.745258)
Supplement: Supplementary file 1 [file DataSheet_1.pdf]

Table S1 The features retained after each feature dimension reduction were as follows:

	Modality (features number)	Univariate Cox regression	Spearman's and Pearson's correlation analysis	LASSO
DFS	CT and MRI (5589)	269	43	17
	CT (2751)	26	12	7
	MRI (2838)	243	32	12
OS	CT and MRI (5589)	750	55	17
	CT (2751)	72	13	8
	MRI (2838)	187	37	16

DFS, disease-free survival; OS, overall survival; LASSO, Least absolute shrinkage and selection operator

Table S2 Radiomic features with non-zero coefficients selected by LASSO analysis.

CT_DFS	**Radiomic features**
Original_shape_Sphericity	Shape
Wavelet_HLH_firstorder_RootMeanSquared	Firstorder
Wavelet_HLL_firstorder_Mean	Firstorder
Log_sigma_3_0_mm_3D_firstorder_Maximum	Firstorder
Wavelet_HHL_glszm_GrayLevelNonUniformityNormalized	GLSZM
Wavelet_HLL_glszm_SizeZoneNonUniformityNormalized	GLSZM
Original_gldm_DependenceNonUniformityNormalized	GLDM
MRI_DFS	
Wavelet_LHH_firstorder_RootMeanSquared	Firstorder
Wavelet_LHH_firstorder_Skewness	Firstorder
Wavelet_HHL_firstorder_Skewness	Firstorder
Wavelet_LLH_glszm_ZoneVariance	GLSZM
Wavelet_HHL_glszm_ZonePercentage	GLSZM
Wavelet_LHL_firstorder_Median	Firstorder
Wavelet_LLL_glcm_ClusterShade	GLCM
Log_sigma_5_0_mm_3D_glcm_Contrast	GLCM
Original_glcm_Correlation	GLCM
Wavelet_HLL_glcm_ClusterProminence	GLCM
Wavelet_HHH_glcm_lmc2	GLCM
Log_sigma_2_0_mm_3D_firstorder_RootMeanSquared	Firstorder
Combined_DFS	
Wavelet_LHH_firstorder_RootMeanSquared	Firstorder
Original_shape_Sphericity	Shape
Wavelet_HLH_firstorder_RootMeanSquared	Firstorder
Wavelet_LHH_firstorder_Skewness	Firstorder
Original_shape_Flatness	Shape
Wavelet_HLL_firstorder_Mean	Firstorder
Original_glcm_Correlation	GLCM
Wavelet_LHH_glszm_SmallAreaEmphasis	GLSZM
Log_sigma_5_0_mm_3D_firstorder_Kurtosis	Firstorder

Log_sigma_2_0_mm_3D_firstorder_RootMeanSquared	Firstorder
Wavelet_HLL_glcm_ClusterProminence	GLCM
Wavelet_HLL_glszm_SizeZoneNonUniformityNormalized	GLSZM
Wavelet_HHL_glszm_ZonePercentage	GLSZM
Wavelet_HHH_glcm_lmc2	GLCM
Wavelet_LLL_glcm_ClusterShade	GLCM
Wavelet_HHL_glszm_GrayLevelNonUniformityNormalized	GLSZM
Original_gldm_DependenceNonUniformityNormalized	GLDM
CT_OS	
Wavelet_LHL_glszm_SmallAreaEmphasis	GLSZM
Wavelet_HLH_gldm_DependenceVariance	GLDM
Log_sigma_5_0_mm_3D_glszm_SizeZoneNonUniformityNormalized	GLSZM
Wavelet_LHL_glszm_SmallAreaLowGrayLevelEmphasis	GLSZM
Wavelet_HHH_glcm_Idn	GLCM
Wavelet_HLH_firstorder_Variance	Firstorder
Log_sigma_5_0_mm_3D_firstorder_90Percentile	Firstorder
Log_sigma_4_0_mm_3D_glrlm_ShortRunHighGrayLevelEmphasis	GLRLM
MRI_OS	
Wavelet_HLH_glszm_ZonePercentage	GLSZM
Wavelet_HLL_firstorder_Skewness	Firstorder
Log_sigma_5_0_mm_3D_firstorder_Skewness	Firstorder
Wavelet_HLL_glcm_ClusterShade	GLSZM
Wavelet_LHL_glszm_SmallAreaLowGrayLevelEmphasis	GLSZM
Wavelet_HLH_glszm_GrayLevelNonUniformityNormalized	GLSZM
Wavelet_LLH_glszm_LowGrayLevelZoneEmphasis	GLSZM
Wavelet_HLH_glszm_GrayLevelNonUniformityNormalized	GLSZM
Wavelet_HLH_glszm_SmallAreaEmphasis	GLSZM
Wavelet_LHL_glcm__ClusterProminence	GLCM
Wavelet_HLH_glszm_LargeAreaLowGrayLevelEmphasis	GLSZM
Log_sigma_2_0_mm_3D_firstorder_90Percentile	Firstorder
Wavelet_LLH_firstorder_Kurtosis	Firstorder
Wavelet_HLL_firstorder_Kurtosis	Firstorder
Wavelet_HLL_glszm_ZonePercentage	GLSZM
Wavelet_LLH_glcm_ClusterProminence	GLCM
Combined_OS	
Wavelet_HLH_glszm_ZonePercentage	GLSZM
Wavelet_LHL_glszm_SmallAreaLowGrayLevelEmphasis	GLSZM
Wavelet_HLH_glszm_GrayLevelNonUniformityNormalized	GLSZM
Wavelet_LHL_glszm_SmallAreaEmphasis	GLSZM
Log_sigma_5_0_mm_3D_firstorder_Skewness	GLSZM
Log_sigma_5_0_mm_3D_glszm_SizeZoneNonUniformityNormalized	GLSZM
Wavelet_HLL_firstorder_Skewness	Firstorder
Wavelet_HLL_glcm_ClusterShade	GLCM
Wavelet_HLH_glszm_SmallAreaEmphasis	GLSZM
Log_sigma_2_0_mm_3D_firstorder_90Percentile	Firstorder
Log_sigma_5_0_mm_3D_firstorder_90Percentile	Firstorder
Log_sigma_3_0_mm_3D_glszm_GrayLevelVariance	GLSZM

Wavelet_HLH_glszm_LargeAreaLowGrayLevelEmphasis	GLSZM
Log_sigma_4_0_mm_3D_glcm__ClusterProminence	GLCM
Wavelet_HHL_firstorder_Kurtosis	Firstorder
Wavelet_HHL_glcm_ClusterProminence	GLCM
Wavelet_LLH_firstorder_Kurtosis	Firstorder

DFS, disease-free survival; OS, overall survival; GLDM, gray-level dependence matrix-based features; GLCM, gray-level co-occurrence features matrix-based features; GLSZM gray-level size zone matrix-based features; GLRLM, gray-level run length matrix-based features.

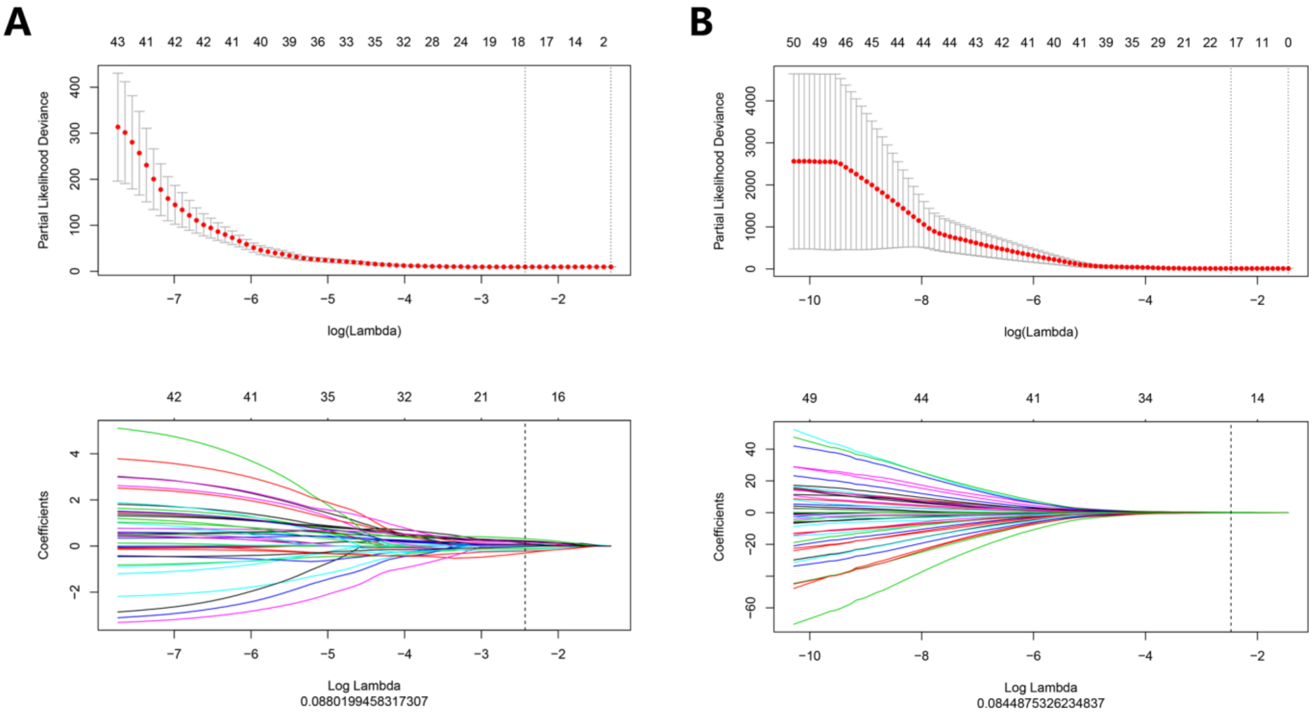

Figure S1. Multimodal (MRI/CT) radiomics feature selection (**A**) Combined_ DFS, (**B**) Combined_ OS. Tuning parameter (Lambda) selection in LASSO involved the use of tenfold cross-validation with minimum criteria. Lambda values (A = 0.088; B = 0.084) were selected as optimal and corresponding vertical lines drawn.
